# Detection of Anatoxins in Human Urine by Liquid Chromatography Triple Quadrupole Mass Spectrometry and ELISA

**DOI:** 10.3390/toxins16030129

**Published:** 2024-03-01

**Authors:** Brady R. Cunningham, Sarah R. Lagon, William A. Bragg, Donna Hill, Elizabeth I. Hamelin

**Affiliations:** 1 Emergency Response Branch, Division of Laboratory Sciences, National Center for Environmental Health, Centers for Disease Control and Prevention, Atlanta, GA 30341, USA; ukz1@cdc.gov (S.R.L.); vtx2@cdc.gov (W.A.B.); 2 Battelle Memorial Institute, Centers for Disease Control and Prevention, 1600 Clifton Road, Atlanta, GA 30333, USA; 3 National Health and Environmental Effects Research, Environmental Protection Agency, 109 T. W. Alexander Drive, Research Triangle Park, NC 27709, USA; hill.donna@epa.gov

**Keywords:** harmful algal blooms, harmful cyanobacterial blooms, anatoxins, LC-MS/MS, clinical, urine, ELISA, dog, mouse

## Abstract

Harmful cyanobacterial blooms are becoming more common and persistent around the world. When in bloom, various cyanobacterial strains can produce anatoxins in high concentrations, which, unlike other cyanobacterial toxins, may be present in clear water. Potential human and animal exposures to anatoxins occur mainly through unintentional ingestion of contaminated algal mats and water. To address this public health threat, we developed and validated an LC-MS/MS method to detect anatoxins in human urine to confirm exposures. Pooled urine was fortified with anatoxin-a and dihydroanatoxin at concentrations from 10.0 to 500 ng/mL to create calibrators and quality control samples. Samples were diluted with isotopically labeled anatoxin and solvent prior to LC-MS/MS analysis. This method can accurately quantitate anatoxin-a with inter- and intraday accuracies ranging from 98.5 to 103% and relative standard deviations < 15%, which is within analytical guidelines for mass spectrometry methods. Additionally, this method qualitatively detects a common degradation product of anatoxin, dihydroanatoxin, above 10 ng/mL. We also evaluated a commercial anatoxin-a ELISA kit for potential diagnostic use; however, numerous false positives were detected from unexposed individual human urine samples. In conclusion, we have developed a method to detect anatoxins precisely and accurately in urine samples, addressing a public health area of concern, which can be applied to future exposure events.

## 1. Introduction

Harmful algal blooms (HABs) and harmful cyanobacterial blooms (HCBs) commonly contaminate freshwater, estuarine, and marine systems and are increasing as a public health threat [1,2]. In freshwater systems, HCBs are caused by cyanobacterial growth and may result in production of numerous toxins including micocytins, anatoxins (ATXs), saxitoxins, and cylindrospermopsins. Microcystins have been the focus of many recent studies, while ATXs have received considerably less attention, despite potent neurotoxic effects [3].

ATXs are highly polar, secondary amine bicyclic alkaloids generated by multiple cyanobacterial genera, including *Dolichospermum*, *Planktothrix*, and *Microcoleus*. These toxins were first identified in Canada in the 1960s, but may have been observed as early as 1878 in Australia [4,5]. Since these initial reports, ATXs have been detected worldwide [6,7,8]. Multiple analogues of ATXs have been reported, with the most common being anatoxin-a (ATX), dihydroanatoxin-a (dhATX), and homoanatoxin-a (HTX) [9,10,11] (Figure S1). Many ATX analogues are environmental degradation by-products, such as hydrolysis or photolysis, metabolic products, or uniquely produced by specific cyanobacterial strains [12,13,14,15,16]. 

Primary exposure to ATXs occurs through the ingestion of contaminated water and algal mats [17,18], In fact, ATXs have also been found in human and animal supplements containing cyanobacteria [19]. When ingested, ATXs act as cholinergic agonists by irreversibly binding muscular and neuronal nicotinic acetylcholine receptors. Reported symptoms of toxic ATX exposure in animal systems include loss of coordination, confusion, muscular spasms, convulsions, and death [5,20,21,22].

ATX exposures have been linked to the deaths of multiple animal species and associated with human illness [5,21,22,23]. A recent report of dog death in the UK occurred only 45 min after direct exposure to ATX-contaminated water, algal mat, and fish [23]. Reports of human intoxication remain anecdotal due to a lack of diagnostic testing. A single report has linked acute ATX poisoning to illness affecting multiple people between 2011 and 2018 following the ingestion of contaminated ascidians, commonly referred to as a sea squirts [6]. ATX was confirmed to be present in the consumed ascidians and closely correlated to the observed symptoms; however, no clinical testing was performed. 

Despite the potential for human illness and animal death, there are inconsistencies in ATX exposure guidance values. The World Health Organization (WHO) has provided provisional reference value limits of 30 µg/L ATX for drinking water and 60 µg/L ATX for recreational water exposure [24], while, in the United States, some states have issued more conservative drinking-water guidance, with limits ranging from 0.1 to 8 µg/L ATX [25,26,27]. Additionally, toxicity data from mouse studies are primarily limited to acute exposures [20,28,29,30,31,32], and ultimately, there is a lack of research on the chronic and sublethal ATXs exposure effects in humans and animals [33].

Currently, there are numerous methods for detecting ATXs in environmental and animal samples using techniques such as enzyme-linked immunoassays (ELISAs), liquid chromatography coupled to tandem mass spectrometry (LC-MS/MS), and LC quadrupole time-of-flight mass spectrometry (LC-QTOF) [15,23,34,35,36,37,38,39]. However, there are no clinically validated methods to detect human ATXs exposure [40]. Here, we tested the suitability of a commercial ELISA for detecting ATXs in human urine and describe an isotope dilution method to detect ATXs in human urine by liquid chromatography coupled with triple quadrupole mass spectrometry (LC-MS/MS). Urine was selected since ATX is highly polar and expected to be excreted quickly. This LC-MS/MS method accurately and precisely quantitates ATX from 10–500 ng/mL and qualitatively detects dhATX above 10 ng/mL. We then used this method to test blank dog urine fortified with ATX to assess whether this method could be used for dog exposures, which currently appear more common than human exposures. Finally, we acquired urine samples from mice acutely exposed to ATX to verify method performance on mammalian exposure samples. The analysis of acutely exposed mouse urine samples measured ATX concentrations from 2748 to 21,450 ng/mL with dhATX detected in each sample. Overall, the LC-MS/MS method described in this manuscript offers a robust diagnostic tool that can be used to confirm suspected human exposures to ATXs and can be used for diagnostic purposes.

## 2. Results

### 2.1. Detection of Anatoxins in Urine by ELISA and Cross-Reactivity

**Figure 1:**
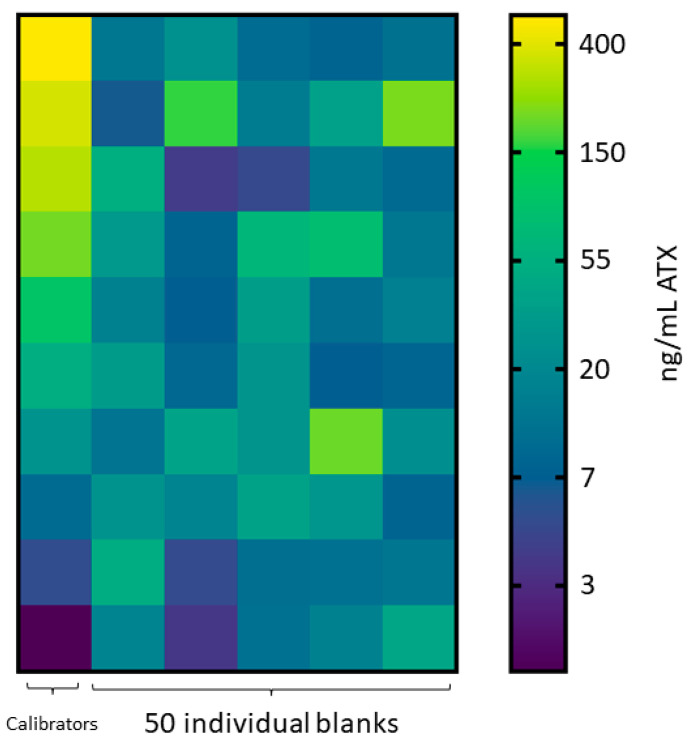
ATX ELISA results. Heat map of individual human urine blank samples diluted 1:20 assessed by ELISA, where the calibration curve ranged 5 to 500 ng/mL ATX equivalents. Scale is natural log transformed.

Anatoxin fortified urine calibrators were evaluated with the commercial anatoxin ELISA kit from 5.00 ng/mL to 500 ng/mL using a 1:20 dilution. The ten calibration curves all produced R^2^ values > 0.98 and maintained calibrator accuracy within 91.1–111%. Fifty blank individual human urine samples were diluted and tested for potential interferences. Twenty-six false positive results above the lowest calibrator concentration (5 ng/mL) were detected (Figure 1), with concentrations ranging from 6.52–214 ng/mL. Further dilutions of the calibrators and individual urine samples at 1:10 and 1:50 still resulted in false positives for 24 and 28 of the 50 individual urine blanks, respectively.

We found cross-reactivity of dhATX at a 1:20 calibrator dilution to be 2.13% in phosphate-buffered saline and 5.65% in pooled human urine matrix for the commercial anatoxin ELISA kit.

### 2.2. LC-MS/MS Method Development

**Figure 2:**
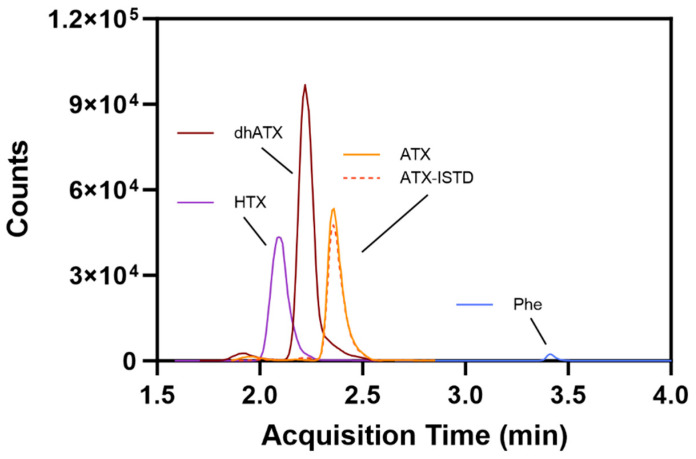
Representative method chromatography displaying separation between homoanatoxin (HTX), dihydroanatoxin (dhATX), anatoxin (ATX), isotopically labeled anatoxin internal standard (ATX-ISTD), and phenylalanine (Phe).

The method developed included two MS transitions (one transition for quantitative and one for qualitative purposes) for each analyte to ensure specificity of detection. The final MS transitions were selected for sensitivity and the absence of background peaks resulting from the urine matrix. Hydrophilic Interaction Liquid Chromatography (HILIC) separation was selected and optimized for a runtime of 5.5 min with the inclusion of ATX and dhATX, while ensuring separation from a potential isobar phenylalanine (Phe) (Figure 2). The sample preparation was a straightforward dilution of 1:20 with acetonitrile:ultrapure water:formic acid (90:10:0.1) *v*/*v* for the subsequent HILIC separation. An isotopically labeled ATX was used as an internal standard to compensate for sample transfer losses and ionization suppression.

### 2.3. LC-MS/MS Method Validation

Quantitation of ATX and dhATX in pooled urine was evaluated for precision and accuracy from 10.0 ng/mL to 500 ng/mL using LC-MS/MS. Three different analysts freshly prepared 22 independent calibration curves with quality control samples (QCs) during the 7-week validation, with no more than 2 curves analyzed per day. The calibration curves all produced R^2^ values > 0.98. Two column lots were used over the course of validation. Interday accuracy of ATX calibrators and the three QCs ranged from 98.5%–103% and 99.4–102%, respectively, while interday accuracy of dhATX calibrators and QCs ranged from 95.7–104% and 97.4–103%, respectively. Interday precision of ATX calibrators and QCs ranged from 3.0–9.9% and 4.4–7.4%, respectively, while interday precision of dhATX calibrators and QCs ranged from 5.4–11.2% and 6.4–10.2% (Table 1). All values fell within the Food and Drug Administration (FDA) guidelines for bioanalytical method validation [41] The intraday accuracy and precision were assessed using additional quality materials, fortified in a single human urine pool at 40, 150, and 350 ng/mL ATX, and were 103–114% and 0.38–6.1%, respectively, while the intraday accuracy and precision for dhATX ranged from 107–113% and 2.42–6.49%, respectively. The method limit of detection (LOD) and limit of quantification (LOQ) for ATX was 2.45 and 8.16 ng/mL, respectively, while the method LOD and LOQ for dhATX was 0.500 and 1.67 ng/mL, respectively [42].

**Figure 3:**
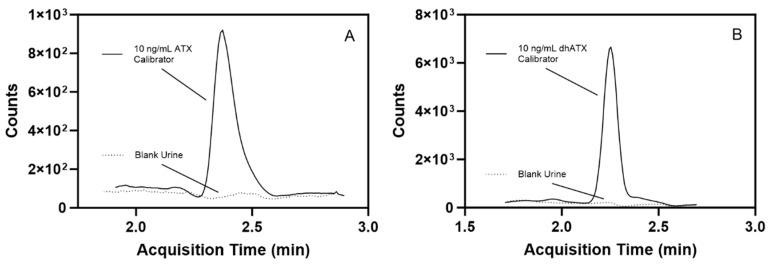
Representative chromatograms showing a low-level, 10 ng/mL fortified ATX calibrator in human urine compared to blank human urine (dotted line) (**A**) and a 10 ng/mL fortified dhATX calibrator in human urine compared to blank human urine (dotted line) (**B**).

**Figure 4:**
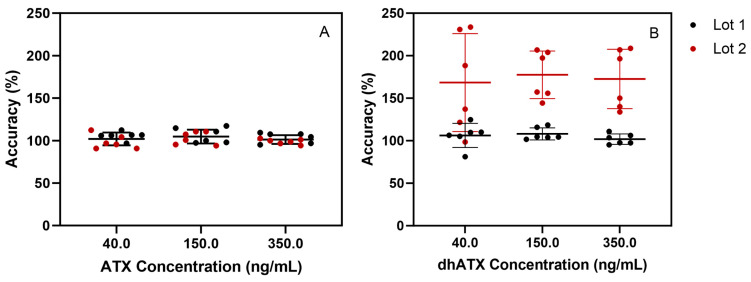
Method accuracy across two separate human urine pools fortified with ATX (**A**) or dhATX (**B**) at three concentrations (40, 150, and 350 ng/mL), run in triplicate over two days of testing (*n* = 6).

Additional experiments for stability, sample dilution capability, method analyte specificity, and accuracy across two pooled human urine lots were assessed. For stability testing, the percent difference of ATX concentration from the initial measurements for ATX varied from −10.9–10.6 for the following conditions: four-month long-term stability, three freeze-thaw cycles, bench-top stability, and processed sample stability (Table S1). For the sample dilution scheme, accuracies of dilutions (100-, 50-, 20-, and 5-fold) ranged from 86.2 to 112%, which also fell within FDA guidelines for bioanalytical methods [41] (Table S2). We further examined method specificity by analyzing 50 individual human urine blank samples, and no background matrix interference peaks were observed at the retention times (RTs) for ATX and dhATX (Figure 3). ATX-ISTD response varied 400-fold in the 50 individual urine samples but was still readily detectible in all samples. Method accuracy in two different pools of human urine was also assessed. Fortified quality materials of ATX and dhATX urine at 40, 150, and 350 ng/mL prepared in the first urine lot produced average accuracies of 102 and 102%, respectively. Fortified quality materials of ATX and dhATX prepared in the second urine lot produced average accuracies of 100 and 173%, respectively (Figure 4).

To assess qualitative method performance for dhATX, data acquired during validation were used to calculate the false positive (FP) and false negative (FN) test rates, creating a confusion matrix in accordance with internal policies. To determine the FP and FN rate, we evaluated a set of 100 samples, of which 60 were fortified with dhATX at varying levels between 10 to 500 ng/mL and 40 were from individual human urine blank samples. From these data, we calculated a true positive rate of 100%, a true negative rate of 100%, a false negative rate of 0%, a false positive rate of 0%, a positive predictive value of 100%, and a negative predictive value of 100%. 

### 2.4. LC-MS/MS Human Urine Matrix Effects by Direct Infusion

**Figure 5:**
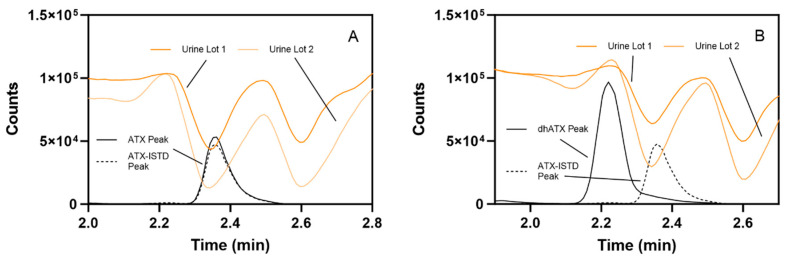
Comparison of (**A**) ATX and ATX-ISTD and (**B**) dhATX and ATX-ISTD ionization in two pooled human urine lots. High ionization suppression occurs during the elution of ATX (black line) and ATX-ISTD (dotted black line), shown as representative chromatograms, in both urine pools (**A**). Slight ionization enhancement for occurs during the elution of dhATX (black line), shown as a representative chromatogram, in both urine pools, while high ionization suppression occurs during the elution of ATX-ISTD (dotted black line), shown as representative chromatogram (**B**).

Comparing matrix effects in two pooled human urine lots, prepared as outlined in the Section 5, revealed that ATX and the ATX internal standard (ATX-ISTD) were highly suppressed in urine. Matrix effects were measured to be 16–46% for ATX and 18–52% for ATX-ISTD. Conversely, dhATX is slightly enhanced, with dhATX matrix effects from 101–105% (Figure 5).

### 2.5. LC-MS/MS Analysis of ATX-Fortified Unexposed Individual Dog Urine Samples

**Figure 6:**
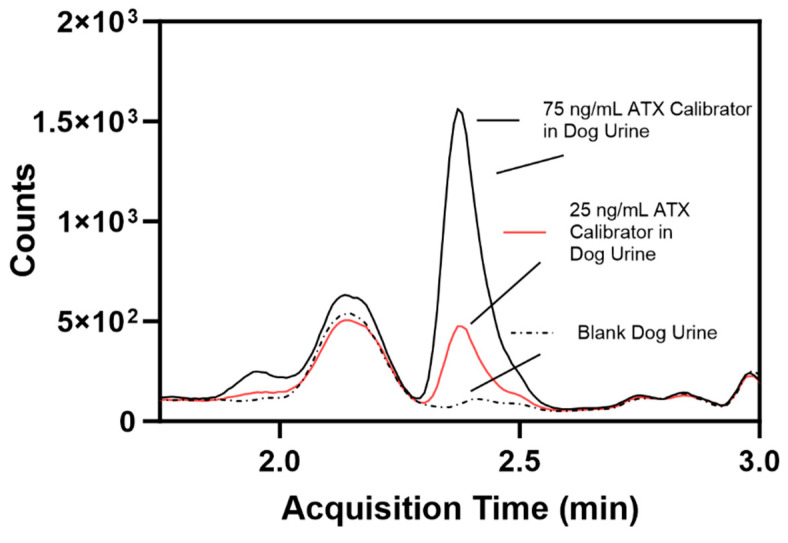
Comparison of individual blank dog urine (dotted line), a 25 ng/mL ATX-fortified dog urine calibrator (red line, calculated limit of quantification), and a 75 ng/mL ATX-fortified dog urine calibrator (black line).

ATX fortified in dog blank urine were run alongside human urine calibrators to determine if this method could be used for dog urine samples, and resulted in accuracies ranging from 101 to 108% and precision ranging from 5.23 to 8.31% (Table 2). The fortified dog urine samples were processed as unknown samples using the calibrators prepared in pooled human urine. A comparison of blank dog urine and 25 ng/mL and 75 ng/mL ATX-fortified dog urine show suppression due to matrix effects (Figure 6). ATX LOD and LOQ in dog urine were determined to be 7.41 and 24.7 ng/mL, respectively, using the Taylor method for calculating LOD [42]. dhATX was detected in each sample indicating positive exposure.

### 2.6. LC-MS/MS Analysis of Acutely Dosed Mouse Samples

Initial analysis of nine mouse samples measured ATX outside of the linear fit range. To ensure accurate quantitation, the mouse samples were diluted 50-fold and re-analyzed. However, even after a 50-fold dilution, one sample concentration was still slightly above the upper reportable limit and was further diluted 100-fold to fit within the dynamic range. ATX was quantitated from 2748 to 21,450 ng/mL and dhATX was detected in all nine acutely dosed mice urine samples (Table 3).

## 3. Discussion

ATXs have been increasingly documented in freshwater systems worldwide and have been responsible for numerous animal exposures and deaths [7]. However, little is currently known about ATX exposure and its effects on humans. In fact, the WHO recognizes that the current ATX toxicological database is not adequate to support the publication of a formal guideline reference value and has only provided provisional guidelines [24]. Analysis from various cases of dog poisoning reveal that ATX exposures can be detected in dog urine [23,43], but there exist no clinical methods to detect potential human exposures in human urine. 

Initially, we investigated the potential for a commercially available ELISA to accurately detect ATXs in human urine. ELISAs have been used to detect ATXs in environmental water, animal tissue, and gut content samples and can provide a cost-effective alternative to methods that require expensive instrumentation and specialized training [44,45]. Our preliminary method development using calibrators and QCs from a single pooled human urine demonstrated acceptable accuracy and precision to indicate this method was fit for purpose. However, this technique encountered problems when assessing blank urine samples. During method development, 24 false positives in 50 individual blank human urine samples were detected (Figure 1). While ELISAs can be more sensitive than LC-MS/MS methods and detect extremely low-level analyte concentrations, ELISAs are a screen and prone to error. The concentrations measured were far above our lowest calibrator (5.00 ng/mL ATX) and in some cases exceeded our top calibrator (500 ng/mL ATX), indicating ELISA error. The concentrations selected for the initial validation (5.00 to 500 ng/mL ATX) were far lower than those measured in the acutely dosed mouse urine samples, which ranged from 2748 to 21,450 ng/mL ATX. Therefore, it is possible that a validation performed using a higher dynamic range may reduce false positives from the urine matrix. For this reason, quantitative results for this commercial ATX ELISA should be evaluated, fully validated before use, and interpreted cautiously due to the potential for false positives.

We further determined this commercial ELISA had low cross-reactivity to dhATX in PBS and urine samples. Since dhATX is a common degradation product of ATX, a biomarker of ATX exposure, and produced by several cyanobacterial strains, this result underpins a major weakness of this commercial ELISA for urine exposure assessment [12,13,14,15,16]. Furthermore, the use of a commercial ELISA kit could underestimate or fail to detect dhATX in clinical and environmental samples. We were not able to test HTX due to limited access to a commercial standard. Because of these ELISA methodological shortcomings, we decided to develop and validate an LC-MS/MS method to detect ATXs in human urine.

The LC-MS/MS method described here can quantitatively detect ATX and qualitatively detect dhATX in human urine. Since exposure data from ATXs are scarce, the only quantitative data points in urine exist from a dog death report in the UK. Using a LC-MS/MS method, the prior study was able to detect 599 and 5495 ng/mL of ATX and dhATX, respectively, from a dog that perished less than one-hour post-exposure [23]. The high concentrations of ATXs in the dog urine indicate that the ATXs are quickly transported through the animal system after acute exposure. Since there are no data on the progression of ATXs in humans after exposure and urine collection may occur more than an hour post-exposure, we elected to develop a more sensitive method. Our method has a quantitative dynamic range for ATX from 10.0 to 500 ng/mL, potentially capturing non-acute exposures and/or delayed urine collection.

While developing this method, we overcame several complications when detecting ATXs in urine. The first and most common problem is chromatographically separating ATX from Phe in urine [46]. Phe presents a common source of interference in urine due to the similar molecular weights of ATX (165.23 *m*/*z*) and Phe (165.19 *m*/*z*), which may not be distinguished with low resolution mass spectrometry, which is used here. In the literature, both reverse phase and HILIC columns have been used in methods detecting ATX; however, reverse phase chromatography is more likely to elute ATX and Phe at similar times [46]. Therefore, HILIC chromatography was selected to separate ATX from Phe by over 60 s while also including dhATX and HTX in the chromatography (Figure 6). To further confirm the differentiation between ATX and Phe, our method includes an isotopically labeled ATX-ISTD, which ensured correct selection of the ATX peak during method development. Continued separation of ATX and Phe was confirmed with an instrument function check before each analysis. HTX was not included in our validation due to lack of a reliably available commercial standard.

The second challenge is analyzing urine samples using minimal sample clean up. ATXs can be extracted using solid phase extraction (SPE), but since ATXs ionize well and exposure concentrations exceed hundreds of ng/mL, we decided to dilute urine samples to minimize sample prep. Diluting urine samples in solvent reduces introduction of unwanted materials in the matrix, but without a more intensive cleanup, we had to ensure our instrumentation remained clean and operating properly. To extend the longevity of our column, we added a guard column. We also visually inspected the source before each run to check for any buildup of material and performed maintenance as needed to ensure minimal background signal. Aside from regular instrument maintenance, we had to update our method during initial characterization due to urine background noise. When first developing this method, the ATX transition 166.1 > 43.1 was selected as the quantification ion due high signal intensity. However, when using urine calibrators, we found that the 166.1 > 43.1 transition retained high signal intensity, but also had higher background noise, especially apparent at lower calibrator levels (Figure S2). Therefore, we switched to using the 166.1 > 91.1 transition as the quantification ion. This transition was relatively less intense than the 43.1 transition, but had much lower background noise, ensuring correct quantification of the lower-level calibrators. Ultimately, a more thorough sample clean up, such as SPE, may be beneficial to avoid excess urine background, but that may be only useful in very low-level exposures, which have yet to be studied.

The third challenge is ATX sensitivity to sunlight and high pH, resulting in rapid decay in the environment [46]. Due to the propensity for ATX to decay, we chose to prep our calibrators and QCs fresh prior to each sample run to ensure ATX degradation did not cause artificially low unknown sample quantification. All unknown samples are kept at −20 °C and in the dark until processed for analysis and then analyzed immediately. We have also tested ATX stability in urine and found minimal change in ATX-fortified urine samples over a variety of conditions (Table S1). Currently, our long-term stability extends to 4 months and longer-term stability is under review.

The fourth challenge encountered was not discovered until we assessed method accuracy by fortifying ATXs at 40, 150, and 350 ng/mL in different lots of pooled human urine. In the first lot of pooled human urine, ATX and dhATX accurately quantitated, both averaging 102% recovery. However, in the second lot of pooled urine, ATX averaged 100% recovery, but dhATX averaged 173% recovery (Figure 4). Since ATXs are highly soluble in water and organic, this was not a solubility issue, and these results indicated that we could not confidently quantitate dhATX in unknown urine samples due to differences in urine matrix effects. To find the root cause of this quantitation disparity between ATX and dhATX, we performed a direct infusion experiment. This experiment identified that ATX and the ATX-ISTD are highly suppressed in urine, while dhATX is slightly enhanced (Figure 5). Since our method uses ATX-ISTD to quantitate ATX and as a reference ISTD for dhATX, the difference in ionization cannot be overcome by solely using ATX-ISTD. To address this limitation, our method detects dhATX qualitatively in human urine and was able to accurately detect positive samples and not detect negative samples in all of the samples tested without false positives or false negatives, as shown in our FP/FN confusion matrix data. To develop a quantitative dhATX method, a dhATX-ISTD or additional sample preparation is required. As of this writing, a dhATX-ISTD is not commercially available. 

The fifth challenge was to determine if this method could be used for detecting exposures of ATXs in dog urine. We used human urine calibrators against fortified dog urine samples, since we could not acquire sufficient quantity of dog urine to create and validate dog urine calibration curves. Upon visual inspection, individual unexposed dog urine samples contain more salts and precipitates than individual human urine samples. Since this method only uses a solvent dilution prior to LC-MS/MS analysis, dog urine samples have increased matrix background effects, leading to ion suppression and a reduction in MS signal, which ultimately increased the method LOD (Figure 6). After fortifying urine from nine individual dogs, ranging from 75 to 150 ng/mL of ATX and dhATX, we calculated an LOD of 7.41 ng/mL and an LOQ of 24.7 ng/mL ATX. Furthermore, dhATX was detected in all samples. Comparatively, the ATX LOD and LOQ in dog urine are three-fold higher than in human urine. Even with this increase, our method can be used to analyze dog urine samples due to the high levels of ATX previously detected, but it is also best practice to create a matrix-matched dog urine calibration curve for future testing [23]. 

Finally, we demonstrated the ability of this method to detect acutely dosed mouse exposures to ATX from a laboratory-controlled EPA study. The analysis of mouse urine samples from this study confirmed the application of this method for acute exposures and revealed a range of 2748 to 21,450 ng/mL ATX and detected dhATX in all nine samples. The interpretation of these data will be discussed in a future manuscript. We also confirmed the ability to dilute and quantitate high concentration samples. Additionally, the mice in this study were only dosed with ATX, and therefore, the presence of dhATX is indicative of ATX transformation and can be an effective biomarker of ATX exposure.

## 4. Conclusions

There is a growing repository of ATX detection methods but currently no clinical methods to detect ATXs in human urine. We tested the ability of a commercially available ATX ELISA to detect ATXs in human urine samples at multiple dilutions and found urine to be an unsuitable matrix at the concentrations evaluated. Furthermore, we have developed and validated an LC-MS/MS ATX detection method providing high specificity and accuracy in human urine samples. We observed enhanced dhATX ionization in human urine samples, while ATX and ATX-ISTD ionization were suppressed. We developed dhATX as a qualitative method because the ionization differences between ATX and dhATX were not overcome by the isotopically labeled ATX-ISTD. Regardless, we present an LC-MS/MS method for detecting ATXs in human urine, which can be quantitatively broadened upon the reliable commercial availability of HTX and the commercial availability of an isotopically labeled dhATX-ISTD.

## 5. Materials and Methods

### 5.1. Chemicals and Materials

ATX, dhATX, HTX, and ^13^C_4_-(+)-anatoxin-a (ATX-ISTD) standards were purchased from Gold Standard Diagnostics (Davis, CA, USA) and stored at −20 °C. Phe (reagent grade, Cat no. P2126-100G) standard was purchased from Sigma-Aldrich (St. Louis, MO, USA) and stored at room temperature. ATX ELISA kits (Cat no. 520060) were obtained from Eurofins Abraxis, currently Gold Standard Diagnostics (Warminster, PA, USA). Ultrapure 18.2 MΩ-cm reagent-grade filtered water was produced in-house using an Aqua Solutions purification system (Jasper, GA, USA). Methanol (HPLC-MS grade, Cat no. 33900HPLC), isopropyl alcohol (HPLC-UV grade, Cat no. 231HPLC99), and acetonitrile (HPLC-UV grade, Cat no. LC015-4) were purchased from Fisher Scientific (Fairlawn, NJ, USA). Optima formic acid (LC-MS grade, Cat no. A117-50) and ammonium formate (LC-MS grade, Cat no. A115-50) were obtained from Fisher Scientific (Fairlawn, NJ, USA). Two mL microcentrifuge tubes (Cat No. 0030123620) were purchased from Eppendorf (Hamburg, Germany). Ninety-six-well PCR plates (Cat No. AB2396) were purchased from Thermo Scientific (Rochester, NY, USA). Acquity BEH Amide 1.7 µm columns (50 × 2.1 mm, Part No. 186004800) and VanGuard BEH Amide 1.7 µm Pre-Column guard columns (Part No. 186004799) were purchased from Waters (Milford, MA, USA). 

Individual and pooled human urine samples were purchased from Tennessee Blood Services (Memphis, TN, USA), which did not meet criteria for human subjects research, as specified in 45-CRF 46.102(f), as they were provided by random, anonymous individuals to represent the general population. Unexposed dog urine samples were supplied by Marshall BioResources (North Rose, NY, USA), and all collection procedures were performed in accordance with the Animal Welfare Act and AVMA Guidelines for the Euthanasia of Animals.

### 5.2. Preparation of Calibrators, Quality Control Samples (QCs), and Fortified Samples for LC-MS/MS and ELISA

ATX, dhATX, and ATX-ISTD stocks (purchased primary stocks and working stocks) and pooled human urine were thawed to room temperature. The 500 ng/mL ATX-ISTD was prepared daily from a 10,000 ng/mL purchased primary stock by diluting an appropriate volume of the stock with sample diluent (90:10:0.1 *v*/*v* acetonitrile/ultrapure water/formic acid, e.g., 10 µL of 10,000 ng/mL into 190 µL diluent). To prepare fresh calibrators and QC samples each day of testing, pooled urine was first centrifuged at 3500× *g* for 5 min to pellet salts. A daily 1000 ng/mL working stock solution was then prepared by fortifying urine in a 2 mL microcentrifuge tube with 5000 ng/mL ATX and dhATX purchased stock standards. The 1000 ng/mL working stock solution was then added to appropriate volumes of pooled urine in 2 mL microcentrifuge tubes such that the following final ATX and dhATX concentrations (ng/mL) were achieved for a final volume of 200 µL: 500, 400, 300 (QC), 200, 100 (QC), 75, 50, 25 (QC), 20, 10, and 0 (blank). Prior to plating, unknown samples were also centrifuged at 3500× *g* for 5 min to pellet salts. 

For LC-MS/MS analysis, 5 µL of 500 ng/mL ATX-ISTD was pipetted to each reaction well of the 96-well PCR plate, followed by 5 uL of calibrators, QCs, or samples to the reaction wells. Each well was further diluted with 95 µL sample diluent (prepared fresh daily, 90:10:0.1 acetonitrile:ultrapure water:formic acid). The plate was heat-sealed, vortexed briefly to mix (≤5 s), briefly centrifuged (≤450× *g* for ≤30 s), and analyzed immediately on the LC-MS/MS system. 

Fortified samples of ATX and dhATX were prepared at 40, 150, and 350 ng/mL from two separate 1000 ng/mL working stocks corresponding to the two independent pooled human urine lots, which were made fresh just prior to sample preparation. Accuracy testing was repeated twice over two test days.

Individual dog urine samples (*n* = 9) were fortified at 75, 100, 125, and 150 ng/mL with ATX and used to assess method sensitivity in non-human biological samples, prepared as outlined for accuracy testing samples. The LOD and LOQ for dog urine samples was determined by using the standard deviation of the fortified samples averaged over nine individual urine samples [42].

### 5.3. Detection of Anatoxins in Urine by ELISA

ELISA kit materials and reagents were allowed to come to room temperature prior to all procedures. Procedures were performed according to manufacturer instructions. Briefly, enzyme conjugate and antibody were reconstituted in 3 mL of the supplied diluent (prepared at a 1:10 dilution) and vortexed. Calibrators, as described above, and blank individual urine dilutions were performed by pipetting 45 µL PBS to the supplied microliter plate followed by the addition of 5 µL calibrator or sample. Following sample dilution/plating, 50 µL reconstituted enzyme conjugate and 50 µL of reconstituted antibody serum were added to each reaction well. The plate was then sealed with foil and allowed to incubate at 25 °C for 1 h on a ThermoMixer C (Hauppauge, NY, USA) set to 800 rpm for 30 s followed by 2 min of no shaking. All wells were rinsed with 250 µL of the provided wash buffer (prepared at a 1:5 dilution in ultrapure water) four times before 100 µL of color solution was added to each reaction well. The plate was re-sealed and incubated for an additional 30 min at 25 °C (shaking parameters stated above). Following this final incubation step, 100 µL of stop solution was pipetted to each well. Absorbances at 450 nm were immediately read on a BioTek Synergy Neo2 microplate spectrophotometer (Santa Clara, CA, USA). Gen5 software version 3.12 was then used to interpolate concentrations from the standard curve. ELISAs were conducted at the following calibrator/individual urine sample dilutions: 1:10, 1:20, 1:50, and 1:100 using PBS. Concentrations above the lowest calibrator (5 ng/mL) were considered positive for ATXs.

### 5.4. ELISA Cross Reactivity

Duplicate calibration curves for dhATX were prepared alongside duplicate ATX calibration curves at the following concentrations: 0, 5, 10, 50, 100, 200, 300, 400, and 500 ng/mL. Cross reactivity was assessed in both water (PBS) and pooled human urine matrix. Following calibrator and sample preparation and plating procedures as outlined above, absorbances were immediately read at 450 nm, and concentrations were calculated using Gen5 software. Subsequent analysis was run in GraphPad Prism (Boston, MA, USA), where IC50s were generated and cross-reactivities calculated. Cross-reactivity of each analyte was calculated with respect to ATX (percent cross-reactivity = (IC50 (half-maximal inhibitory concentration) ATX/IC50 Analyte) × 100) [47].

### 5.5. Chromatography Conditions

ATX and dhATX were separated on an Agilent 1290 liquid chromatography system (CA, USA) using a Waters Acquity BEH Amide column with a Vanguard Pre-Column (1.7 um, 50 × 2.1 mm, Milford, MA, USA) maintained at 30 °C (Figure 6). The mobile phase (A) comprised 90:10:0.1 (*v*/*v*) ultrapure water/100 mM ammonium formate/formic acid and (B) comprised 98:2:0.1 (*v*/*v*) acetonitrile/100 mM ammonium formate/formic acid, which were both made fresh daily. The gradient elution profile was programmed to a 500 µL/min flow rate beginning at 98% B from 0.00 to 1.00 min, decreasing to 75% B to 4.00 min, increasing to 98% B to 4.50, and held at 98% B to 5.50 min. The injection volume was set to 5 µL with a needle rinse step of 5 s using 60:20:20:0.1% (*v*/*v*) isopropyl alcohol/methanol/acetonitrile/formic acid.

### 5.6. Mass Spectrometry Conditions

ATX, dhATX, HTX, and Phe were detected using an Agilent 6495b triple quadrupole mass spectrometer (Santa Clara, CA, USA) with a jet stream electrospray ion spray source operating in positive ion mode. The following parameters were used for all transitions: MS mode, DMRM; gas temp, 150 °C; gas flow, 15 L/min; nebulizer, 50 psi; sheath gas temp, 400 °C; sheath gas flow, 12 L/min; capillary (positive), 2500 V; high pressure RF (positive), 150 V; and low-pressure RF (positive), 80 V.

### 5.7. Ion Suppression Direct Infusion Experiment

Urine matrix effects on ATX and dhATX were evaluated in two human urine lots as described previously [48]. Briefly, a post-column syringe pump was attached to the LC-MS/MS system via a tee to the column effluent. The syringe operated continuously at a constant rate of 25 µL/min delivering either 100 ng/mL of ATX or dhATX in mobile phase B solvent. Ion suppression for each analyte was compared by injecting a 5 µL aliquot of sample diluent and two different human urine lots, as described previously.

Ionizations differences were calculated for ATX, ATX-ISTD, and dhATX by first locating the peak RT for each analyte. Then, using the peak RT for each analyte, the associated ionization counts in the direct infusion experiment were determined for solvent and both urine lots. Finally, the percent ionization difference was calculated for each analyte by dividing the ionization counts in each urine lot by the ionization counts in solvent.

### 5.8. LC-MS/MS ATX Quantitative Data Analysis

Two mass transitions were identified (Table 4), and MassHunter version 10.0 (Agilent, Santa Clara, CA, USA) software was used to integrate data peaks. Standard curves were generated by plotting the ratio of each calibrator peak area over the ATX-ISTD area with 1/x weighting. Individual and pooled urine samples fortified with ATX were interpolated from the standard curves. Inter-day percent accuracies were established by comparing the mean of measured concentration to the theoretical concentration of calibrator and QC samples across 7 weeks of testing. Intra-day percent accuracies were established by comparing the mean of the measured concentration to the theoretical concentration of fortified urine samples.

### 5.9. LC-MS/MS dhATX Qualitative Data Analysis

Qualitative tests report a positive or negative result. To ensure appropriate method performance, the quality control samples prepared above were run for dhATX. A positive result for dhATX is determined by assessing if a potential peak falls within the following criteria: transition 1 ion count, transition 2 ion count, confirmation ion ratio, RT, and relative RT (RRT). Additionally, fortified and blank individual samples were tested to determine the following rates: diagnostic sensitivity or True Positive Rate − TP/(TP + FN), diagnostic specificity or True Negative Rate − TN/(TN + FP), False Negative Rate − FN/(FN + TP), False Positive Rate − FP/(FP + TN), positive predictive value − TP/(TP + FP) × 100, and negative predictive value − TN/(TN + FN) × 100. TP is the total number of positive samples in the blinded set that were identified as positive and were positive (true positive), TN is the total number of negative samples in the blinded set that were identified as negative and were negative (true negative), FP is the total number of samples there were identified as positive but were negative (false positive), and FN is the total number of samples there were identified as negative but were positive (false negative) [49].

### 5.10. LC-MS/MS Method Stability

Analyte stability was assessed after initially measuring two QCs (25 and 100 ng/mL of ATX and dhATX) and then measuring again after the sets of QC materials went through four separate processes; (i) 4 months stored at −20 °C to determine long-term stability, (ii) left at room temperature overnight to assess short-term, bench-top stability, (iii) remeasured processed samples after sitting overnight, and (iv) three freeze-thaw cycles to mimic sample handling conditions.

### 5.11. LC-MS/MS Dilution Scheme Evaluation

Multiple sample dilutions were evaluated to prepare samples that were above the reportable range following an initial analysis. To assess dilution linearity, pooled human urine was fortified with ATX standard at a concentration of 2000 ng/mL and then diluted 100-, 50-, 20-, and 5-fold. Dilutions were performed in triplicate and assessed.

### 5.12. Mouse Exposure and Urine Collection

Male and female CD-1 mice, 10–12 weeks old, were purchased from Charles Rivers Laboratories (Raleigh, NC, USA) and shipped to USEPA (Research Triangle Park, NC, USA). The mice were housed, same sex, two–three per cage with heat-treated, laboratory-grade pine shavings (Northeast Products, Warrensburg, NY, USA) and fed 5001 Rodent Chow (Purina, St. Louis, MO, USA) and filtered (5 µm) municipal tap water (NC, USA) ad libitum. This study was conducted in accordance with a protocol approved by the USEPA Center for Public Health and Environmental Assessment Institutional Animal Care and Use Committee. Animals were housed in a facility accredited by the Association for Assessment and Accreditation of Laboratory Animal Care and maintained at 20–22 °C, 45–55% humidity, and a 12:12 h photoperiod (06:00–18:00 EST). After a 5-day acclimation period, mice were randomly assigned to a treatment group using a random number generator. These mice were used for preliminary dose-finding and the urine sent to the Centers for Disease Control and Prevention (CDC) (Atlanta, GA, USA) laboratory was collected from four female and five male mice dosed orally with a single dose of either 2, 4, or 7 mg/kg ATX (Cawthron Institute, Nelson, New Zealand). The urine was collected post-dosing at either 45 or 90 min. The urine was immediately collected and placed on dry-ice in the dark until it was transferred to a −80 °C freezer. The urine samples were kept frozen and in the dark until shipped overnight on dry ice to the CDC. The ATX standard was quantitated prior to dosing and was measured as 112% of expected concentration with 2.6% of dhATX present (Greenwater Laboratories, Palatka, FL, USA), likely due to degradation during storage in a −20 °C freezer > 1 year.

## Figures and Tables

**Table 1:** Quantitative method validation results for ATX and dhATX. Interday (*n* = 22) percent accuracy and relative standard deviation (RSD) for calibrators and quality controls (QCs) and intraday (*n* = 3) percent accuracy and RSD for fortified samples in two pooled human urine lots.

		ATX (ng/mL)	Average (ng/mL)	RSD (%)	Accuracy (%)	dhATX (ng/mL)	Average (ng/mL)	RSD (%)	Accuracy (%)
Interday Calibrators		500	496	2.98	99.3	500	490	5.29	98.1
		400	403	3.87	101	400	406	5.91	102
		200	199	5.93	99.4	200	200	7.36	100
		75.0	77.4	8.28	103	75.0	77.8	10.6	104
		50.0	49.2	8.17	98.5	50.0	50.9	10.5	102
		20.0	20.2	5.72	101	20.0	20.1	8.97	100
		15.0	14.9	7.70	99.3	15.0	14.9	8.11	99.0
		10.0	9.86	9.87	98.6	10.0	9.53	10.9	95.3
Interday QCs	High	300	298	6.39	99.4	300	295	10.5	98.5
	Med.	100	102	4.60	102	100	103	6.61	103
	Low	25.0	24.8	7.41	99.4	25.0	25.0	10.2	100
Intraday Samples	Matrix Lot 1	350	362	6.07	103	350	374	3.56	107
		150	171	3.10	114	150	169	6.49	113
		40.0	42.4	0.38	106	40	42.9	2.42	107
	Matrix Lot 2	350	355	1.24	101	350	741	4.03	204
		150	164	1.81	110	150	304	3.51	203
		40.0	37.7	3.36	94.3	40	87.0	8.94	218

**Table 2:** Results for ATX-fortified individual dog urine samples showing average (avg), standard deviation (Stdev), percent accuracy, and relative standard deviation (RSD) (*n* = 9).

ATX-Fortified Dog Urine Samples				
	75 ng/mL ATX Fortification	100 ng/mL ATX Fortification	125 ng/mL ATX Fortification	150 ng/mL ATX Fortification
Avg (ng/mL)	80.4	108	132	151
Stdev (ng/mL)	5.60	5.74	6.47	8.38
Accuracy (%)	107	108	106	101
RSD (%)	5.23	5.30	6.13	8.31

**Table 3:** ATX concentrations in urine from mice orally administered ATX.

Treatment (Sex)	ATX Mouse Dose (mg/kg)	ATX Mouse Dose (Total µg)	Collection Time after Dosing (min)	ATX Concentration (ng/mL)	dhATX Presence
ATX (F)	2	65	45	4120	Detect
ATX (F)	2	74	45	17,400	Detect
ATX (M)	2	83	45	16,900	Detect
ATX (F)	4	138	45	18,200	Detect
ATX (M)	4	158	45	15,700	Detect
ATX (M)	4	172	45	3540	Detect
ATX (F)	7	230	45	2750	Detect
ATX (M)	7	289	90	20,900	Detect
ATX (M)	7	305	90	21,500	Detect

**Table 4:** Characteristic fragment ions utilized during dynamic multiple reaction monitoring (DMRM). ^a^ Settings should be optimized per instrument.

Analyte Name	Transitions	Collision Energy (V) ^a^	Ion Function
ATX	166.1 > 91.1	30	Quantitation ion
	166.1 > 77.1	46	Confirmation ion
ATX-ISTD	170.0 > 93.1	30	Internal standard ion
dhATX	168.1 > 43.1	38	Qualitative Transition 1 ion
	168.1 > 67.1	34	Qualitative Transition 2 ion

## Data Availability

The data presented in this study are available in this article.

## Supplementary Materials

The following supporting information can be downloaded at: https://www.mdpi.com/article/10.3390/toxins16030129/s1, Figure S1: Structures of ATX, dhATX, and HTX; Figure S2: Comparison between ATX transitions; Table S1: ATX stability; Table S2: Dilutions for ATX-fortified human urine samples.

## References

[1] Huisman J., Codd G.A., Paerl H.W., Ibelings B.W., Verspagen J.M.H., Visser P.M. (2018). Cyanobacterial Blooms. Nat. Rev. Microbiol..

[2] Brooks B.W., Lazorchak J.M., Howard M.D.A., Johnson M.V.V., Morton S.L., Perkins D.A.K., Reavie E.D., Scott G.I., Smith S.A., Steevens J.A. (2016). Are Harmful Algal Blooms Becoming the Greatest Inland Water Quality Threat to Public Health and Aquatic Ecosystems?. Environ. Toxicol. Chem..

[3] Plata-Calzado C., Prieto A.I., Cameán A.M., Jos A. (2022). Toxic Effects Produced by Anatoxin-a under Laboratory Conditions: A Review. Toxins.

[4] Gorham P.R., McLachlan J., Hammer U.T., Kim W.K. (1964). Isolation and Culture of Toxic Strains of Anabaena Flos-Aquae (Lyngb.) de Bréb. SIL Proc. 1922–2010.

[5] Francis G. (1878). Poisonous Australian Lake. Nature.

[6] Biré R., Bertin T., Dom I., Hort V., Schmitt C., Diogène J., Lemée R., de Haro L., Nicolas M. (2020). First Evidence of the Presence of Anatoxin-A in Sea Figs Associated with Human Food Poisonings in France. Mar. Drugs.

[7] Colas S., Marie B., Lance E., Quiblier C., Tricoire-Leignel H., Mattei C. (2021). Anatoxin-a: Overview on a Harmful Cyanobacterial Neurotoxin from the Environmental Scale to the Molecular Target. Environ. Res..

[8] Lovin L.M., Brooks B.W. (2020). Global Scanning of Anatoxins in Aquatic Systems: Environment and Health Hazards, and Research Needs. Mar. Freshw. Res..

[9] Méjean A., Paci G., Gautier V., Ploux O. (2014). Biosynthesis of Anatoxin-a and Analogues (Anatoxins) in Cyanobacteria. Toxicon.

[10] James K.J., Furey A., Sherlock I.R., Stack M.A., Twohig M., Caudwell F.B., Skulberg O.M. (1998). Sensitive Determination of Anatoxin-a, Homoanatoxin-a and Their Degradation Products by Liquid Chromatography with Fluorimetric Detection. J. Chromatogr. A.

[11] Skulberg O.M., Skulberg R., Carmichael W.W., Andersen R.A., Matsunaga S., Moore R.E. (1992). Investigations of a Neurotoxic Oscillatorialean Strain (Cyanophyceae) and Its Toxin. Isolation and Characterization of Homoanatoxin-a. Environ. Toxicol. Chem..

[12] Méjean A., Dalle K., Paci G., Bouchonnet S., Mann S., Pichon V., Ploux O. (2016). Dihydroanatoxin-a Is Biosynthesized from Proline in Cylindrospermum Stagnale PCC 7417: Isotopic Incorporation Experiments and Mass Spectrometry Analysis. J. Nat. Prod..

[13] Conklin K.Y., Stancheva R., Otten T.G., Fadness R., Boyer G.L., Read B., Zhang X., Sheath R.G. (2020). Molecular and Morphological Characterization of a Novel Dihydroanatoxin-a Producing Microcoleus Species (Cyanobacteria) from the Russian River, California, USA. Harmful Algae.

[14] Kust A., Méjean A., Ploux O. (2020). Biosynthesis of Anatoxins in Cyanobacteria: Identification of the Carboxy-Anatoxins as the Penultimate Biosynthetic Intermediates. J. Nat. Prod..

[15] James K.J., Crowley J., Hamilton B., Lehane M., Skulberg O., Furey A. (2005). Anatoxins and Degradation Products, Determined Using Hybrid Quadrupole Time-of-Flight and Quadrupole Ion-Trap Mass Spectrometry: Forensic Investigations of Cyanobacterial Neurotoxin Poisoning. Rapid Commun. Mass Spectrom..

[16] Faassen E.J., Harkema L., Begeman L., Lurling M. (2012). First Report of (Homo)Anatoxin-a and Dog Neurotoxicosis after Ingestion of Benthic Cyanobacteria in The Netherlands. Toxicon.

[17] Pawlik-Skowrońska B., Toporowska M., Rechulicz J. (2012). Simultaneous Accumulation of Anatoxin-a and Microcystins in Three Fish Species Indigenous to Lakes Affected by Cyanobacterial Blooms. Oceanol. Hydrobiol. Stud..

[18] Al-Sammak M.A., Hoagland K.D., Cassada D., Snow D.D. (2014). Co-Occurrence of the Cyanotoxins BMAA, DABA and Anatoxin-a in Nebraska Reservoirs, Fish, and Aquatic Plants. Toxins.

[19] Solter P.F., Beasley V.R. (2013). Phycotoxins. Haschek and Rousseaux’s Handbook of Toxicologic Pathology.

[20] US EPA (2015). Health Effects Support Document for the Cyanobacterial Toxin Anatoxin-A.

[21] Codd G.A., Edwards C., Beattie K.A., Barr W.M., Gunn G.J. (1992). Fatal Attraction to Cyanobacteria?. Nature.

[22] Fredrickson A., Richter A., Perri K.A., Manning S.R. (2023). First Confirmed Case of Canine Mortality Due to Dihydroanatoxin-a in Central Texas, USA. Toxins.

[23] Turner A.D., Turner F.R.I., White M., Hartnell D., Crompton C.G., Bates N., Egginton J., Branscombe L., Lewis A.M., Maskrey B.H. (2022). Confirmation Using Triple Quadrupole and High-Resolution Mass Spectrometry of a Fatal Canine Neurotoxicosis Following Exposure to Anatoxins at an Inland Reservoir. Toxins.

[24] World Health Organization (2020). Cyanobacterial Toxins: Anatoxin-a and Analogues.

[25] (2016). Anatoxin-a and Drinking Water Info Sheet.

[26] Hardy F.J., Johnson A., Hamel K., Preece E. (2015). Cyanotoxin Bioaccumulation in Freshwater Fish, Washington State, USA. Environ. Monit. Assess..

[27] Zeise L. (2022). Recommendations for Acute Notification Levels for Anatoxin-A, Cylindrospermopsin, Microcystins and Saxitoxins.

[28] Valentine W.M., Schaeffer D.J., Beasley V.R. (1991). Electromyographic Assessment of the Neuromuscular Blockade Produced in Vivo by Anatoxin-a in the Rat. Toxicon.

[29] Stevens D.K., Krieger R.I. (1988). Analysis of Anatoxin-a by GCIECD. J. Anal. Toxicol..

[30] Fawell J.K., Mitchell R.E., Hill R.E., Everett D.J. (1999). The Toxicity of Cyanobacterial Toxins in the Mouse: II Anatoxin-A. Hum. Exp. Toxicol..

[31] Wonnacott S., Swanson K.L.L., Albuquerque E.X.X., Huby N.J.S.J.S., Thompson P., Gallagher T. (1992). Homoanatoxin: A Potent Analogue of Anatoxin-A. Biochem. Pharmacol..

[32] Puddick J., van Ginkel R., Page C.D., Murray J.S., Greenhough H.E., Bowater J., Selwood A.I., Wood S.A., Prinsep M.R., Truman P. (2021). Acute Toxicity of Dihydroanatoxin-a from *Microcoleus autumnalis* in Comparison to Anatoxin-A. Chemosphere.

[33] Christensen V.G., Khan E. (2020). Freshwater Neurotoxins and Concerns for Human, Animal, and Ecosystem Health: A Review of Anatoxin-a and Saxitoxin. Sci. Total Environ..

[34] Osswald J., Rellán S., Gago A., Vasconcelos V. (2007). Toxicology and Detection Methods of the Alkaloid Neurotoxin Produced by Cyanobacteria, Anatoxin-A. Environ. Int..

[35] Dimitrakopoulos I.K., Kaloudis T.S., Hiskia A.E., Thomaidis N.S., Koupparis M.A. (2010). Development of a Fast and Selective Method for the Sensitive Determination of Anatoxin-a in Lake Waters Using Liquid Chromatography-Tandem Mass Spectrometry and Phenylalanine-d 5 as Internal Standard. Anal. Bioanal. Chem..

[36] Roy-Lachapelle A., Solliec M., Sinotte M., Deblois C., Sauvé S. (2015). High Resolution/Accurate Mass (HRMS) Detection of Anatoxin-a in Lake Water Using LDTD-APCI Coupled to a Q-Exactive Mass Spectrometer. Talanta.

[37] John N., Baker L., Ansell B.R.E., Newham S., Crosbie N.D., Jex A.R. (2019). First Report of Anatoxin-a Producing Cyanobacteria in Australia Illustrates Need to Regularly up-Date Monitoring Strategies in a Shifting Global Distribution. Sci. Rep..

[38] Cevallos-Cedeño R.E., Quiñones-Reyes G., Agulló C., Abad-Somovilla A., Abad-Fuentes A., Mercader J.V. (2022). Rapid Immunochemical Methods for Anatoxin-a Monitoring in Environmental Water Samples. Anal. Chem..

[39] Rutkowska M., Płotka-Wasylka J., Majchrzak T., Wojnowski W., Mazur-Marzec H., Namieśnik J. (2019). Recent Trends in Determination of Neurotoxins in Aquatic Environmental Samples. TrAC-Trends Anal. Chem..

[40] Drijvers J.M., Awan I.M., Perugino C.A., Rosenberg I.M., Pillai S. (2017). The Enzyme-Linked Immunosorbent Assay: The Application of ELISA in Clinical Research. Basic Science Methods for Clinical Researchers.

[41] U.S. Department of Health and Human Services (2018). Bioanalytical Method Validation: Guidance for Industry.

[42] Taylor J.K. (1987). Quality Assurance of Chemical Measurements.

[43] Puschner B., Pratt C., Tor E.R. (2010). Treatment and Diagnosis of a Dog with Fulminant Neurological Deterioration due to Anatoxin-a Intoxication. J. Vet. Emerg. Crit. Care.

[44] Anderson B., Voorhees J., Phillips B., Fadness R., Stancheva R., Nichols J., Orr D., Wood S.A. (2018). Extracts from Benthic Anatoxin-Producing Phormidium Are Toxic to 3 Macroinvertebrate Taxa at Environmentally Relevant Concentrations. Environ. Toxicol. Chem..

[45] Stepanova N., Nikitin O., Latypova V., Kondratyeva T. (2018). Cyanotoxins as a Possible Cause of Fish and Waterfowl Death in the Kazanka River (Russia). Proceedings of the International Multidisciplinary Scientific Geoconference Surveying Geology and Mining Ecology Management, SGEM.

[46] Furey A., Crowley J., Hamilton B., Lehane M., James K.J. (2005). Strategies to Avoid the Mis-Identification of Anatoxin-a Using Mass Spectrometry in the Forensic Investigation of Acute Neurotoxic Poisoning. J. Chromatogr. A.

[47] Zeck A., Weller M.G., Bursill D., Niessner R. (2001). Generic Microcystin Immunoassay Based on Monoclonal Antibodies against Adda. Analyst.

[48] Annesley T.M. (2003). Ion Suppression in Mass Spectrometry. Clin. Chem..

[49] Trevethan R. (2017). Sensitivity, Specificity, and Predictive Values: Foundations, Pliabilities, and Pitfalls in Research and Practice. Front. Public Health.

