# Supplementary material for: Detection of Anatoxins in Human Urine by Liquid Chromatography Triple Quadrupole Mass Spectrometry and ELISA
Source: Toxins (Basel). 2024 Mar 1;16(3):129. doi: 10.3390/toxins16030129 (PMC10975466; doi:10.3390/toxins16030129)
Supplement: Supplementary file 1 [file toxins-16-00129-s001.zip › Figure S1.pdf]

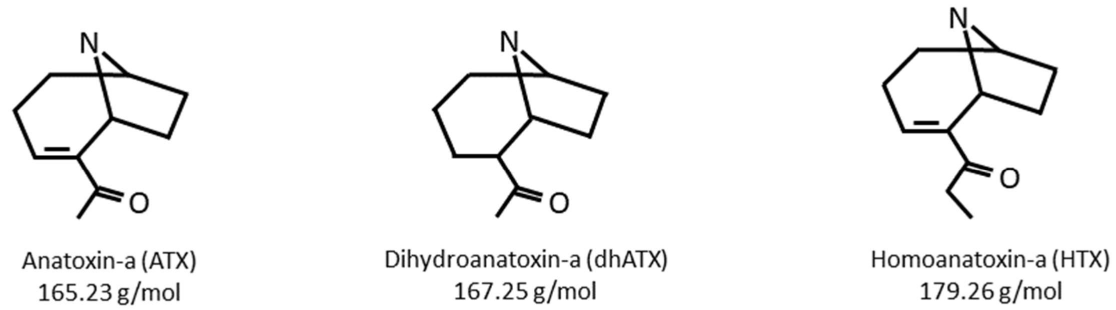

Figure S1. Structures of ATX, dhATX, and HTX.
