# Supplementary material for: Detection of Anatoxins in Human Urine by Liquid Chromatography Triple Quadrupole Mass Spectrometry and ELISA
Source: Toxins (Basel). 2024 Mar 1;16(3):129. doi: 10.3390/toxins16030129 (PMC10975466; doi:10.3390/toxins16030129)
Supplement: Supplementary file 1 [file toxins-16-00129-s001.zip › Table S1.pdf]

toxins

MDPI

Table S1. Method stability testing parameters and results (ng/mL) for ATX; measured results were calculated from means of fortified urine samples prepared in triplicate.

ATX Stability						
	**QC Concentration**	**Initial Measurement**	**Long-term Stability**	**Bench-top Stability**	**Processed Sample Stability**	**Three Freeze-thaw Cycles**
	25.0	24.7	23.0	23.0	27.3	22.0
% difference from initial measurement	---	---	-6.85	-6.90	10.6	-10.9
	100	96.3	89.4	99.6	103	94.9
% difference from initial measurement	---	---	-7.16	3.4	6.47	-1.44
