# Supplementary material for: Detection of Anatoxins in Human Urine by Liquid Chromatography Triple Quadrupole Mass Spectrometry and ELISA
Source: Toxins (Basel). 2024 Mar 1;16(3):129. doi: 10.3390/toxins16030129 (PMC10975466; doi:10.3390/toxins16030129)
Supplement: Supplementary file 1 [file toxins-16-00129-s001.zip › Table S2.pdf]

toxins

Table S2. Results of dilution scheme evaluation for ATX-fortified pooled human urine samples showing average (avg), standard deviation (Stdev), percent accuracy, and relative standard deviation (RSD). Fortified samples were prepared in triplicate.

Dilutions for ATX-fortified Human Urine Samples				
	2000 ng/mL ATX Spike 1:100 Dilution	2000 ng/mL ATX Spike 1:50 Dilution	2000 ng/mL ATX Spike 1:20 Dilution	2000 ng/mL Spike ATX 1:5 Dilution
Avg (ng/mL)	17.2	44.7	98.0	358
Calculated Conc (ng/mL)	1723	2235	1959	1790
Stdev (ng/mL)	1.21	2.96	6.26	8.19
RSD (%)	7.02	6.62	6.39	2.29
Accuracy (%)	86.2	112	98.0	89.5
